# Combating COVID-19: MVA Vector Vaccines Applied to the Respiratory Tract as Promising Approach Toward Protective Immunity in the Lung

**DOI:** 10.3389/fimmu.2020.01959

**Published:** 2020-08-07

**Authors:** Reinhold Förster, Henrike Fleige, Gerd Sutter

**Affiliations:** ^1^Institute of Immunology, Hannover Medical School, Hanover, Germany; ^2^Cluster of Excellence RESIST (EXC 2155), Hannover Medical School, Hanover, Germany; ^3^Division of Virology, Institute for Infectious Diseases and Zoonoses, Ludwig-Maximilians-Universität München, Munich, Germany; ^4^German Center for Infection Research, Partner Site Munich, Munich, Germany

**Keywords:** MVA, COVID-19, SARS-CoV-2, vaccine, lung, BALT

## Abstract

The lung is the vital target organ of coronavirus disease 2019 (COVID-19) caused by infection with severe acute respiratory syndrome coronavirus 2 (SARS-CoV-2). In the majority of patients the most active virus replication seems to be found in the upper respiratory tract, severe cases however suffer from SARS-like disease associated with virus replication in lung tissues. Due to the current lack of suitable anti-viral drugs the induction of protective immunity such as neutralizing antibodies in the lung is the key aim of the only alternative approach—the development and application of SARS-CoV-2 vaccines. However, past experience from experimental animals, livestock, and humans showed that induction of immunity in the lung is limited following application of vaccines at peripheral sides such as skin or muscles. Based on several considerations we therefore propose here to consider the application of a Modified Vaccinia virus Ankara (MVA)-based vaccine to mucosal surfaces of the respiratory tract as a favorable approach to combat COVID-19.

## Introduction

Like skin and intestinal tract the lung stays in close contact with the living environment and is therefore potentially exposed to air-borne pathogens including SARS-CoV-2. However, in contrast to skin and intestine that harbor large amounts of effector immune cells and lymphoid tissues, the non-inflamed lung is largely devoid of both components rendering this organ particularly vulnerable for airborne infections.

Several subsets of immune cells populate the skin. Antigen-presenting Langerhans cells reside in the epidermis while dendritic cells, macrophages, and various populations of T cells occupy the dermis (1). Likewise, T cells reside in the intestinal epithelial layer and the intestinal lamina propria is crowded by a plethora of immune cells including T cells, macrophages, dendritic cells, and in particular plasma cells. The latter secrete large amounts of immunoglobulins of the IgA isotype into the mucus overlaying the intestinal epithelium and into the gut lumen. Furthermore, large parts of the intestinal mucosa are decorated with secondary lymphoid organs such as Peyer's patches, cryptopatches, or isolated lymphoid follicles that all have been shown to contribute to the induction and maintenance of systemic as well as mucosal immune responses (2).

## Bronchus-Associated Lymphoid Tissue—Balt

Lungs of healthy adults however are largely devoid of lymphoid structures and immune cells are sparsely found in the mucosal layers lining the trachea and the bronchial tree, in the alveolar space but not in the alveolar interstitium. Adaptive immune responses of the lung are primarily initiated and maintained in bronchial and mediastinal lymph nodes that drain the lung but usually not within the lung tissue (3). Furthermore, following application of vaccines at peripheral sides such as skin or muscles, immune protection of the lung is limited (4).

Interestingly, children and adolescents—but not healthy adults—frequently harbor tertiary lymphoid tissue structures within the lung (5). These are positioned around bronchi and next to large blood vessels and are known as bronchus-associated lymphoid tissue (BALT). BALT is part of the mucosa-associated lymphoid tissue that is composed of B cell follicles that are encircled by a para-follicular area rich of T cells and antigen-presenting cells (6). The relevance of BALT for the induction of protective immunity was first demonstrated in splenectomized lymphotoxin-alpha-deficient mice that lack all secondary lymphoid organs. Following repeated stimulation with lipopolysaccharide these mice developed BALT that allowed the induction of protective immunity following otherwise lethal challenges with influenza virus (7).

## Modified Vaccinia Virus Ankara—Backbone of Successful Vector Vaccines

MVA is a highly attenuated orthopoxvirus that was growth adapted to avian cells and lost its ability to replicate in mammalian hosts. Historically, it had been safety tested in more than 100,000 persons during the smallpox eradication campaign in Germany in the 1970s. Today, MVA serves as basis for licensed vaccines against smallpox and monkeypox in Europe, Canada and the United States (European Medicines Agency, EMA/490157/2013; US Food and Drug Administration, FDA STN 125678/24.09.2019) (8). Moreover, MVA expressing recombinant antigens have been shown to be safe and immunogenic in many human subjects in various clinical studies including more than 1300 infants in Africa and immune compromised patients (9–11). Recent studies using a recombinant MVA expressing the spike protein of the Middle East respiratory syndrome coronavirus (MERS-CoV), a close relative to SARS-CoV-2, revealed safety and immunogenicity upon intramuscular application in a phase 1 trial (12). The same vector vaccine, when co-administered by intranasal and intramuscular inoculations, solidly protected dromedary camels against respiratory tract virus replication following challenge with MERS-CoV (13).

## MVA and Balt

During the last decade we studied extensively the ability of MVA to induce BALT and subsequent immune responses in mice. The single intranasal application of MVA in mice induced long lasting formation of BALT within 6–8 days. Extensive immunological characterization, including two-photon microscopy, revealed that MVA-induced BALT acts as a general priming site for T cells against antigens that reach the lower respiratory tract. Furthermore, following intranasal but not intramuscular application, MVA led to the accumulation of activated CD8 effector T cells in the lung (6, 14). Due to lack of experimental data it is currently unknown whether other viral vectors used for vaccination also induce BALT after delivery via the respiratory route or whether even mRNA-based vaccines would have the potential to induce BALT once delivered into the lung.

**Figure 1:**
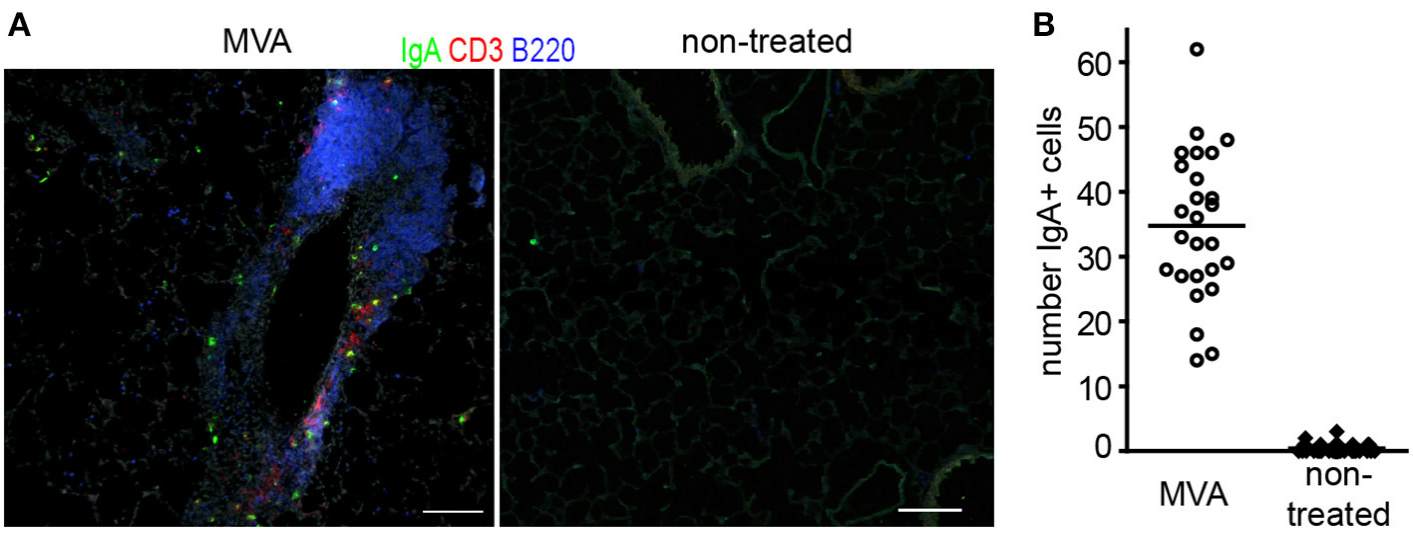
Representative sections **(A)** and quantitative analysis of IgA+ plasma cells in lung sections of mice **(B)** 12 day after intranasal application of 10^7^ IU non-recombinant MVA or left untreated; Lungs were snap frozen and 8 μm thick sections were prepared. Sections were stained with the antibodies indicated. Micrographs were taken with a 10x lense and numbers of IgA^+^ cells were determined per micrograph; dots = micrographs analyzed; *n* = 3 mice per group, 6–10 micrographs per lung analyzed, bars, 100 μm.

Arguably, the induction of plasma cells secreting neutralizing antibodies of the IgA isotype would be most suited to prevent air-borne lung infections. We therefore studied to what degree intranasal application of MVA led to induction of IgA plasma cells in the lung. To that end mice received a single dose of MVA or were left untreated. Twelve days later lungs were analyzed by histology for the presence of BALT as well as plasma cells. While untreated mice carried on average 0.4 IgA^+^ plasma cells per mm^2^ tissue section this value increased 87-fold to 34.7 IgA^+^ plasma cells/mm^2^ in MVA-treated mice (Figure 1). Although likely, additional experiments will be required to prove that the IgA plasma cells identified actually secrete anti-MVA antibodies.

These findings are in agreement with observations of others identifying ovalbumin-specific IgA antibodies in the bronchoalveolar lavage fluid in mice intranasally immunized with MVA expressing ovalbumin (15). Others showed in non-human primates that aerosol immunization with recombinant MVA induced specific IgA in the genital tract (16, 17) or T cells specific for the transgene in the lung (18). Furthermore, it has been shown that intranasal application of MVA expressing a secreted form of the bovine herpesvirus 1 (BoHV-1) glycoprotein D protects against BoHV-1 infection in a rabbit model (19). Application of MVA vector vaccines via the respiratory route has been shown to be safe in general (16) but respiratory adverse effects have been reported after intramuscular priming and inhalative boosting with MVA expressing the 85A antigen of Mycobacterium tuberculosis (20).

## Conclusion

The profound production of lymphoid tissue that acts as a priming side for immune responses in the lung including induction of T cells as well as IgA-secreting plasma cells renders the local application of MVA encoding the SARS-CoV-2 spike protein a promising approach for preclinical and clinical studies aiming at the prevention of COVID-19.

## Data Availability Statement

All datasets generated for this study are included in the article/supplementary material.

## Ethics Statement

This animal study was reviewed and approved by Niedersächsisches Landesamt für Verbraucherschutz und Lebensmittelsicherheit, LAVES.

## Author Contributions

HF conducted the research. RF conceived the study and analyzed the data. RF and GS wrote the manuscript. All authors contributed to the article and approved the submitted version.

## Conflict of Interest

The authors declare that the research was conducted in the absence of any commercial or financial relationships that could be construed as a potential conflict of interest.
